# Proteinase Activated Receptor 1 Mediated Fibrosis in a Mouse Model of Liver Injury: A Role for Bone Marrow Derived Macrophages

**DOI:** 10.1371/journal.pone.0086241

**Published:** 2014-01-27

**Authors:** Yiannis N. Kallis, Christopher J. Scotton, Alison C. MacKinnon, Robert D. Goldin, Nicholas A. Wright, John P. Iredale, Rachel C. Chambers, Stuart J. Forbes

**Affiliations:** 1 Department of Hepatology, St. Mary's Hospital Campus, Imperial College London, London, United Kingdom; 2 Centre for Inflammation & Tissue Repair, University College London, London, United Kingdom; 3 MRC Centre for Inflammation Research, The Queen's Medical Research Institute, University of Edinburgh, Edinburgh, United Kingdom; 4 Department of Histopathology, St. Mary's Hospital Campus, Imperial College London, London, United Kingdom; 5 Barts Cancer Institute, Barts and The London School of Medicine and Dentistry, London, United Kingdom; 6 MRC Centre for Regenerative Medicine, The Queen's Medical Research Institute, University of Edinburgh, Edinburgh, United Kingdom; University of Birmingham, United Kingdom

## Abstract

Liver fibrosis results from the co-ordinated actions of myofibroblasts and macrophages, a proportion of which are of bone marrow origin. The functional effect of such bone marrow-derived cells on liver fibrosis is unclear. We examine whether changing bone marrow genotype can down-regulate the liver's fibrotic response to injury and investigate mechanisms involved. Proteinase activated receptor 1 (PAR1) is up-regulated in fibrotic liver disease in humans, and deficiency of PAR1 is associated with reduced liver fibrosis in rodent models. In this study, recipient mice received bone marrow transplantation from PAR1-deficient or wild-type donors prior to carbon tetrachloride-induced liver fibrosis. Bone marrow transplantation alone from PAR1-deficient mice was able to confer significant reductions in hepatic collagen content and activated myofibroblast expansion on wild-type recipients. This effect was associated with a decrease in hepatic scar-associated macrophages and a reduction in macrophage recruitment from the bone marrow. *In vitro*, PAR1 signalling on bone marrow-derived macrophages directly induced their chemotaxis but did not stimulate proliferation. These data suggest that the bone marrow can modulate the fibrotic response of the liver to recurrent injury. PAR1 signalling can contribute to this response by mechanisms that include the regulation of macrophage recruitment.

## Introduction

Fibrosis is a consequence of chronic liver disease and may lead to cirrhosis and liver failure. Hepatic stellate cells (HSC) and activated myofibroblasts are the predominant liver cell-types that produce collagen in response to chronic injury. Recent studies have clearly shown that the bone marrow (BM) can contribute to hepatic myofibroblast populations in murine models of liver injury and in patients with liver fibrosis [1]–[4]. The BM contributes to other non-parenchymal cell-types in the liver such as macrophages, which are known to regulate both scar formation and resolution [5]. Rats depleted of macrophages develop less liver fibrosis, and impairment of monocyte chemotaxis and activation leads to less liver fibrosis and HSC activation [6]–[8]. Moreover, BM-derived cells, such as macrophages, may also aid liver recovery by assisting in fibrous matrix degradation via the production of matrix metalloproteinases and anti-inflammatory cytokines [9]–[11].

Coagulation cascade proteinases, such as thrombin, play key roles in inflammation and fibrosis in multiple organs including the liver [12]–[16]. Hepatic fibrosis progression is correlated with pro-coagulant status in humans with chronic viral hepatitis and in murine models of liver fibrosis [16], [17]. Indeed antagonism of thrombin has been shown to prevent fibrosis progression in these models [14], [16]. Thrombin signals via cellular proteinase activated receptors (PAR), predominantly PAR1, which is widely expressed on fibroblasts, macrophages, platelets, and endothelium [18]. PAR1 is localised to activated HSCs and macrophages in cirrhotic livers in humans and is up-regulated on HSC and macrophage activation *in vitro* [19]–[23]. Pharmacological antagonism of PAR1 reduces portal fibrosis in the rat bile duct ligation model of cholestatic liver injury, and the hepatic scarring response to carbon tetrachloride (CCl_4_) injury is reduced in PAR1-knockout mice (PAR1(−/−)) [22], [24]. Additionally, PAR1-deficient mice exposed to steatohepatitic injury exhibit lower levels of liver inflammation [25]. Genetic polymorphisms of PAR1 may influence fibrosis progression in humans with hepatitis C infection [26].

The precise functional contribution of the BM to the liver's fibrotic response to injury has not been clearly defined, and the mechanisms involved require further clarification. The role of PAR1 signalling on BM-derived cells, in particular that on BM-derived macrophages, is yet to be elucidated. By performing bone marrow transplantation (BMT) experiments in a murine model of fibrotic liver injury using mice deficient in functional PAR1, we sought to investigate the mechanisms by which PAR1 signalling in BM-derived cells, in particular in macrophages, may influence liver fibrosis.

## Methods

### Mouse models and induction of liver fibrosis

All animal work was approved by the British Home Office (project licence no. 70/5956) and carried out under its procedural and ethical guidelines. Mice were housed in self-contained isolation units with free access to drinking water and standard chow. PAR1(−/−) mice (C57/BL6 background), which lack functional PAR1 and wild-type (WT) littermates were bred in house. The production (involving backcrossing on to a C57/BL6 background >10 generations) and phenotype of this mouse have been previously described [27]. In contrast to humans, mouse platelets do not express PAR1 so coagulation status is unaltered in these knockout mice [28]. 4–8 week-old female PAR1(−/−) mice (n = 8) and age-matched female WT littermates (n = 8) received 6 weeks CCl_4_ (Sigma-Aldrich, Harlan, UK) via intra-peritoneal injection three times a week to induce liver fibrosis. CCl_4_ diluted in sunflower oil was administered at the following concentrations: week 1 1∶31, week 2 1∶15, week 3 1∶7, weeks 4–6 1∶3. A total volume of 0.1 mL was given with each intra-peritoneal injection. Mouse livers were harvested 72 h after the last CCl_4_ injection.

To examine whether BM genotype influences liver fibrosis, 4–6 week-old female recipient C57/BL6 mice (Harlan, Bicester, UK, n = 8 per group) received BMT from age-matched PAR1(−/−) or C57/BL6 WT male donors. BM cells were obtained from the pelvis, femur and tibia of donor mice in cooled PBS with 2% fetal calf serum (FCS) (n = 4 per group). Female recipient mice, maintained on acidified water, were myelo-ablated by whole body irradiation (total 10 Gray in two doses), and injected with unfractionated male BM in PBS via the tail vein. BMT was sex-mismatched to enable BM-derived cells to be identified using the Y chromosome. After four weeks to allow for BM reconstitution, liver fibrosis was induced with the same CCl_4_ regimen described above.

### Immunohistochemistry and Y chromosome in situ hybridisation

Liver tissue was routinely processed and 5 µm-thick sections cut. Fibrosis was assessed by Picrosirius Red (BDH Laboratory, Poole, UK) staining for collagen. Immunohistochemistry was performed for myofibroblasts (αSMA), macrophages (F4/80), endothelium (endomucin) and PAR1 using the primary antibodies and antigen retrieval steps shown in Table 1. Appropriate secondary and tertiary antibody layers were used to develop signal for light microscopy or immunofluorescence. Where detection of the Y chromosome was required, *in situ* hybridisation was subsequently performed. Liver tissue was permeabilised in 1 mol/L sodium thiocyanate at 80°C (10 min), digested in 0.4% pepsin in 0.1 mol/L hydrochloric acid at 37°C (7 min), fixed in 4% paraformaldehyde, then dehydrated. A FITC-labelled Y-chromosome paint (Star-FISH, Cambio, Cambridge, UK), was added, sections denatured at 60°C (10 min), then incubated overnight at 37°C. Sections were nuclear-counterstained with DAPI.

**Table 1:** Details of primary antibodies used for immunohistochemistry.

Cell	Antigen	Dilution	Incubation	Manufacturer	Antigen Retrieval
Myofibroblast	αSMA	1∶2000	1 h	Sigma-Aldrich, Dorset, UK	SCMW pH6 5 min
Macrophage*	F4/80	1∶40	1 h	Insight Biotech, Wembley, UK	0.1% chymotrypsin 37°C 15 min
Macrophage**	F4/80	1∶200	1 h	Abcam, Cambridge, UK	SCMW pH6 5 min
Endothelium	Endomucin	1∶200	1 h	Santa Cruz Biotech, CA, USA	SCMW pH6 20 min
PAR1	PAR1	1∶1000	Overnight 4°C	n/a§	SCMW pH6 20 min

αSMA, α-smooth muscle actin; PAR1, proteinase activated receptor 1; SCMW, sodium citrate buffer (2.94 g/L) microwave,

* used for total hepatic macrophage counting with light microscopy.

** used for fluorescence immunodetection of macrophages in conjunction with *in situ* hybridisation for Y chromosome.

§ a kind gift from Dr Eleanor Mackie, Melbourne, Australia.

### Liver microscopy and image analysis

Sections were viewed by light microscopy (Nikon Eclipse E600, DXM 1200F camera, Nikon, Kingston-upon-Thames, UK) or fluorescent microscopy (Zeiss Axioplan, Carl Zeiss, Welwyn Garden City, UK, with a triple bandpass filter). Cell counting was performed blinded on 10 consecutive randomly-selected ×400 magnification fields unless otherwise stated. To quantify the histological distribution of hepatic collagen and activated myofibroblasts, digital image analysis was performed blinded on an average of 12 randomly-selected ×100 fields from each section and analysed using a Zeiss AxioVert 200M microscope and AnalySIS software (Soft Imaging System Inc, NY).

### Assessment of hepatic gene expression by real time RT-PCR

Total RNA was extracted from frozen, powdered liver using TRIzol reagent as per the manufacturer's protocol. Random hexamers were used as primers for the reverse transcription of 1 µg RNA using the Applied Biosystems kit (Applied Biosystems, Foster City, CA), according to the manufacturer's instructions. Complementary DNA (cDNA) was synthesised from 1 µg of RNA per sample using the GeneAmp RT-PCR kit (Applied Biosystems). Real time RT-PCR was performed using Platinum SYBR Green qPCR SuperMix UDG (Invitrogen) on a LightCycler 1.5 Real-time Detection System (Roche, Welwyn Garden City, UK) and analysed using LightCycler Real-time PCR Detection System Software Version 3.5. 2 µL of cDNA were added to make a 20 µL final volume PCR mix. The nucleotide sequences of primers used for messenger RNA (mRNA) target gene analysis are shown in Table 2. All samples were normalised against hypoxanthine guanine phosphoribosyl transferase housekeeping gene expression. To facilitate a quantitative comparison, individual sample values were normalised against respective mean WT expression.

**Table 2:** Primer nucleotide sequences used for real-time quantitative RT-PCR.

	Target Gene	Primer
Mouse	COL1A1	**For**: 5′ TCGTGGCTTCTCTGGTCTC 3′
		**Rev**: 5′ CCGTTGAGTCCGTCTTTGC 3′
Mouse	HPRT	**For**: 5′ TCATTATGCCGAGGATTTGG 3′
		**Rev**: 5′ ACAGAGGGCCACAATGTGAT 3′

Col 1A1, collagen Iα1; HPRT, hypoxanthine guanine phosphoribosyl transferase.

### Cell Lines and Culture

Primary BM-derived macrophages (BMDM) were isolated by flushing the femurs and tibias of 8–12 week-old C57/BL6 mice and then matured in macrophage selective media for 7–9 days as previously described [5]. A human monocyte cell line, THP-1, and a murine macrophage cell line, RAW 264.7, (both American Tissue Culture Collection, Rockville, MD) were also used. For details of cell culture and media see Table 3.

**Table 3:** Details of cell culture media.

Cell-type	Culture Medium
BMDM	DMEM, 20% L929 conditioned media^16^, 10% FCS, 1% penicillin, 1% streptomycin
THP-1	RPMI-1640 medium, 10% FCS
RAW264.7	DMEM, 10% FCS, 1% L-glutamine, 100 IU/mL penicillin, 100 µg/mL streptomycin

BMDM, primary mouse bone marrow-derived macrophage; DMEM, dulbecco's modified eagle medium; FCS, fetal calf serum.

### In vitro monocyte migration assay

BMDM's (2×10^5^ cells in 200 µL) were washed in serum-free medium and left to settle overnight in the upper chamber of 12 mm transwell inserts containing an 8 µm pore size polycarbonate membrane (Corning, NY). Thereafter, 500 µL of serum-free medium, containing varying concentrations of SFLLRN (Sigma-Aldrich, UK), a synthetic PAR1 ligand, was added to the lower chamber. Monocyte chemoattractant protein (MCP1, 100 ng/mL, R&D Systems, Minneapolis, MN) or 10% FCS were used as positive controls and serum-free medium as a negative control (n = 2 per group, experiments in duplicate). After 4 h incubation at 37°C, non-migrated cells were wiped off the upper surface of the membrane and cells attached to the under-surface were methanol-fixed and stained. Migrated cells were counted from four randomly-selected fields using a ×20 objective. Migration in THP-1 cells was also assessed this way (3 h incubation time).

### In vitro macrophage proliferation assay

RAW 264.7 cells and BMDM's were serum-starved for 16 h, seeded at densities of 6.25×10^3^ or 2×10^5^ cells/well in 96-well plates, then incubated in various concentrations of SFLLRN at 37°C for 24 h & 72 h, respectively. FCS and serum-free medium acted as positive and negative controls, respectively. (n = 2, experiments in duplicate). To quantify proliferation, cells were then incubated with MTT (3-[4,5-dimethylthiazol-2yl]-2,5-diphenyl tetrazolium bromide, 0.4 mg/mL, Sigma-Aldrich) for 3 h, formazan crystals eluted with DMSO and absorbance measured at 560 nm.

### Statistics

Statistical analysis employed the t test, Mann Whitney test and the one-way ANOVA with Dunnett's post-test, for parametric and non-parametric data, respectively, where appropriate (GraphPad Prism, La Jolla, CA). A p value cut-off of 0.05 was taken as significant and results presented as mean +/− SEM.

## Results

### PAR1 is up-regulated during chronic liver injury and is abundantly expressed by hepatic macrophages

**Figure 1:**
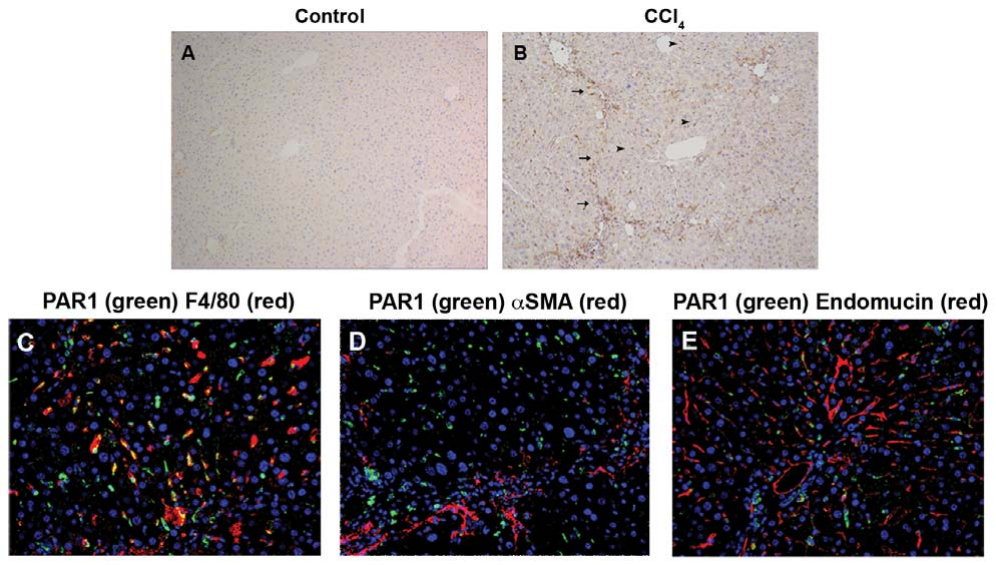
Hepatic PAR1 is up-regulated during liver injury and is expressed on liver macrophages. (A–B) In wild-type mice, there is a marked up-regulation of hepatic PAR1 (brown, ×100) after carbon CCl_4_ injury on cells along the hepatic scars (black arrows) and within the lobule itself (black arrowheads). (C–E) PAR1 (green) is commonly co-localised to macrophages (C, F4/80, red), but only occasionally to myofibroblasts (D, αSMA, red) and hepatic endothelium (E, endomucin, red). (Fluorescence images at ×200 magnification, nuclei in blue.)

Iterative intra-peritoneal CCl_4_ administration induced hepatocyte necrosis, inflammatory cell infiltration and the formation of thick fibrous septae. We report that PAR1 is abundantly expressed along the septal scars and within the hepatic lobule, with expression increasing markedly after liver injury (figure 1). Within the septal scars, co-staining for PAR1 and F4/80 was commonly found. In contrast, only a minority of αSMA-positive septal myofibroblasts and hepatic endothelial cells expressed PAR1.

### Absence of PAR1 signalling leads to diminished liver fibrosis after CCl_4_ injury

**Figure 2:**
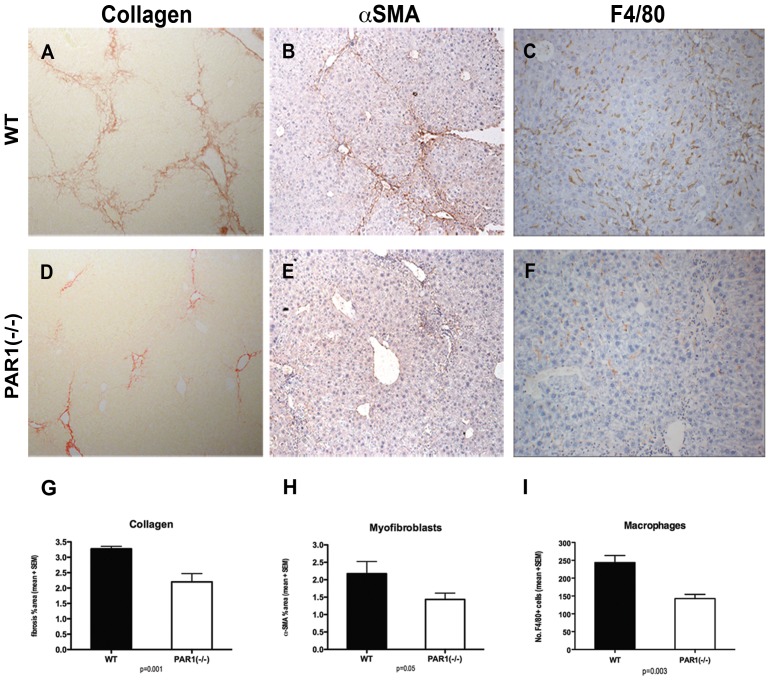
Loss of PAR1 diminishes the hepatic fibrotic response to CCl_4_ liver injury. PAR1(−/−) mice acquire significantly less hepatic collagen (images A&D, sirius red, ×100; graph G), fewer activated myofibroblasts (images B&E, αSMA, brown, ×100; graph H) and fewer hepatic macrophages (images C&F, F4/80, brown, ×200; graph I) after CCl_4_ injury compared to control WT mice (WT, A–C). (graphs show mean + SEM, p values as shown.)

**Figure 3:**
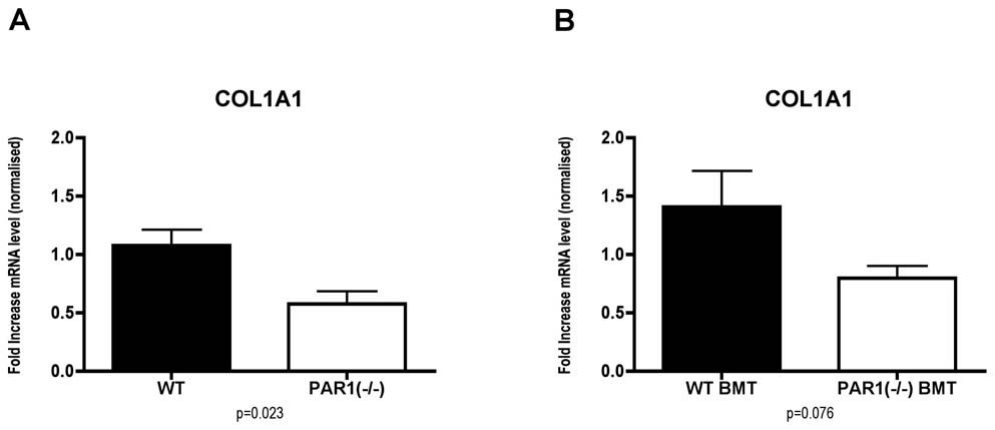
Hepatic collagen I mRNA expression *in vivo*. A considerable reduction in COL1A1 gene expression was found in PAR1(−/−) mice subjected to carbon tetrachloride (CCl_4_) liver injury compared to WT controls (A). A corresponding reduction in COL1A1 gene expression of lower magnitude was seen WT mice transplanted with PAR1(−/−) bone marrow (PAR1(−/−) BMT) compared to WT controls transplanted with WT bone marrow (WT BMT) (B). (graphs show mean + SEM, normalised to hypoxanthine guanine phosphoribosyl transferase housekeeping gene expression and against respective controls, p values as shown.)

To examine the role of PAR1 signalling in hepatic fibrosis, PAR1(−/−) mice and WT littermates received 6 weeks CCl_4_, resulting in hepatic inflammation and scarring. PAR1(−/−) mice acquired significantly less liver fibrosis than WT controls with sparser, thinner fibrotic bands, as assessed by histological collagen percentage surface area (2.20+/−0.27% vs. 3.28+/−0.08%, p = 0.001). Additionally, there was a lower density of activated myofibroblasts, assessed by histological percentrage surface area (αSMA+, 1.43+/−0.18% vs. 2.18+/−0.35%, p = 0.05) (figure 2). Analysis at gene expression level confirmed that PAR1(−/−) mice had significantly lower levels of COL1A1 mRNA than WT controls (p = 0.023) (figure 3). This confirms that PAR1 signalling contributes to liver fibrosis in this experimental model.

### Absence of PAR1 signalling is associated with a reduction in scar-associated macrophages after liver injury

Given the abundant expression of PAR1 on hepatic macrophages, we examined whether loss of functional PAR1 during liver injury resulted in differences in the hepatic macrophage population. Analysis revealed significantly fewer macrophages in the scarred livers of PAR1(−/−) mice compared to WT controls (143+/−12 vs. 244+/−20, p = 0.003, figure 2). To ensure that PAR1(−/−) mice were not constitutively deficient in macrophages, the livers of uninjured PAR1(−/−) mice and WT littermates were compared. To minimise sampling error due to lower total cell numbers, 10 consecutive randomly-selected low power rather than high power fields were counted. There were no differences between PAR1(−/−) mice and WTs (mean cell counts 205+/−18 vs. 234+/−12, p = 0.63). The attenuated fibrotic phenotype secondary to PAR1 deficiency is therefore associated with a lower number of hepatic macrophages. We next sought to investigate the specific contribution of BM-derived cells to the hepatic scar-associated cell populations and the mechanisms by which PAR1 signalling might mediate liver injury.

### Attenuation of liver fibrosis in PAR1(−/−) mice: role of the bone marrow

**Figure 4:**
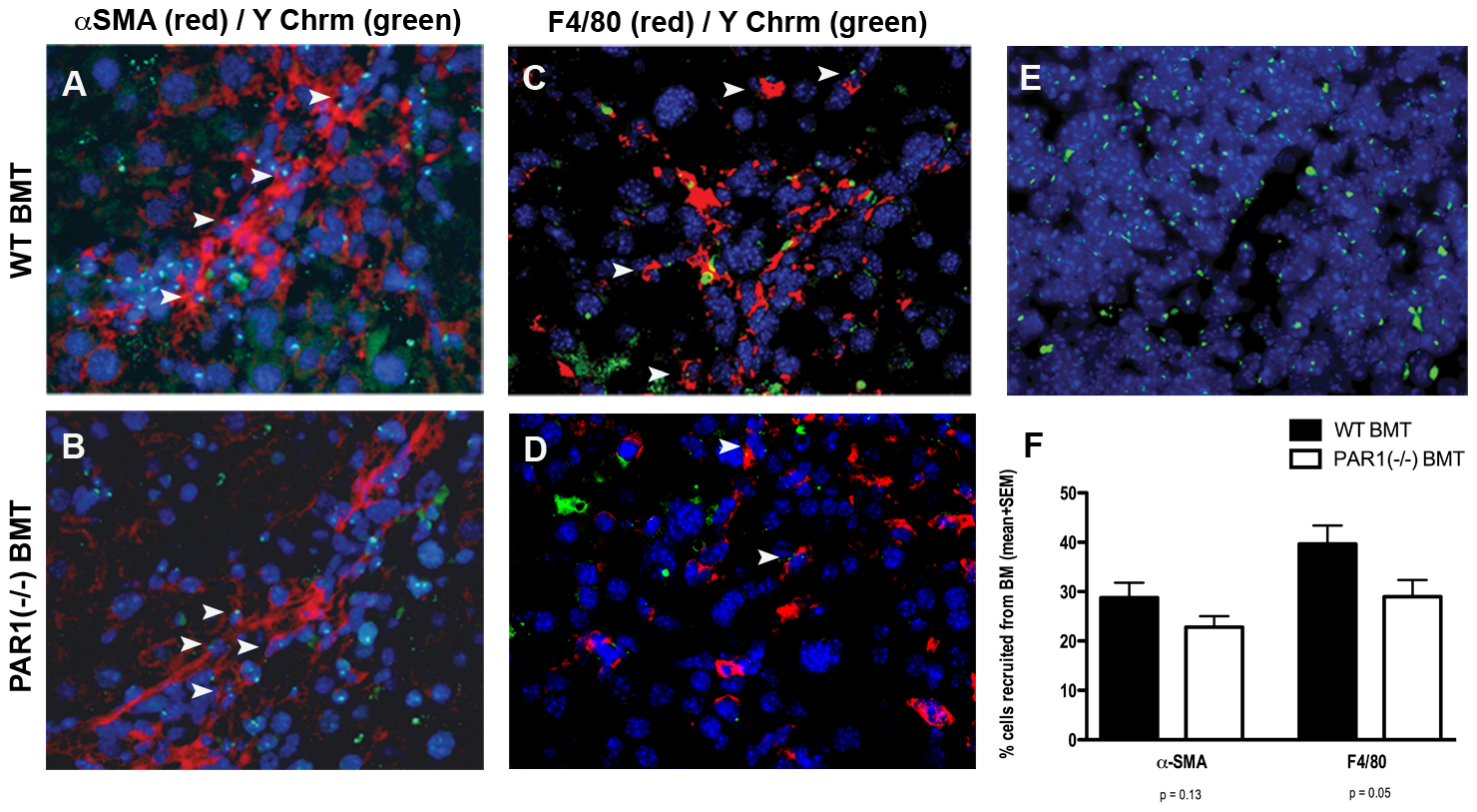
Absence of PAR1 signalling on BM-derived cells is associated with a significant reduction in macrophage recruitment to the injured liver. Female C57/BL6 mice received BMT from male PAR1 knockout donors (PAR1(−/−)BMT, B&D) or from male C57/BL6 WT controls (WTBMT, A&C) before CCl_4_ liver injury. In both groups, BM-derived myofibroblasts (αSMA, red, A&B) and hepatic macrophages (F4/80, red, C&D) are seen (examples indicated by white arrowheads, ×400 magnification). (E) Splenic tissue showing complete haematopoietic reconstitution of recipient mice (female) with male cells, validating the efficacy of the BMT protocol. In all panels BM-derived (male) cells are identified by Y chromosome *in situ* hybridisation (green dot, Y chrm), localised within nuclei (DAPI, blue). (F) Graph showing the relative proportion of hepatic myofibroblasts (αSMA) and macrophages (F4/80) of BM origin. There is a significant reduction of BM-derived macrophage infiltration into the liver with loss of PAR1 signalling. (n = 8 per group, mean + SEM, p values as shown.)

BM cells from PAR1(−/−) mice were transplanted into C57/BL6 WT recipients to ascertain whether modification of BM genotype alters the fibrotic response in the CCl_4_-injured livers of recipient animals. C57/BL6 WT recipients, transplanted with C57/BL6 WT BM, served as controls. Male BM was transplanted into myelo-ablated female recipients to enable tracking of BM-derived cells by Y chromosome *in situ* hybridisation. Successful haematological reconstitution of the recipient animal with donor BM was verified by confirming replacement of recipient splenic tissue with male donor cells in all animals (figure 4). Considerable hepatic engraftment of BM-derived myofibroblasts was seen in recipient animals, consistent with our previous published data (figure 4) [1]. Engraftment rates ranged between 20–30% of total myofibroblast number. Given that nuclei in 5 µm liver sections will not include the Y chromosome approximately one-third of the time, this equates to adjusted engraftment rates of 30–45% [1]. No significant differences were found between the proportional engraftment of myofibroblasts in the WT BMT and PAR1(−/−) BMT groups, suggesting that PAR1 signalling does not play a role in the hepatic migration of these fibrogenic cells.

**Figure 5:**
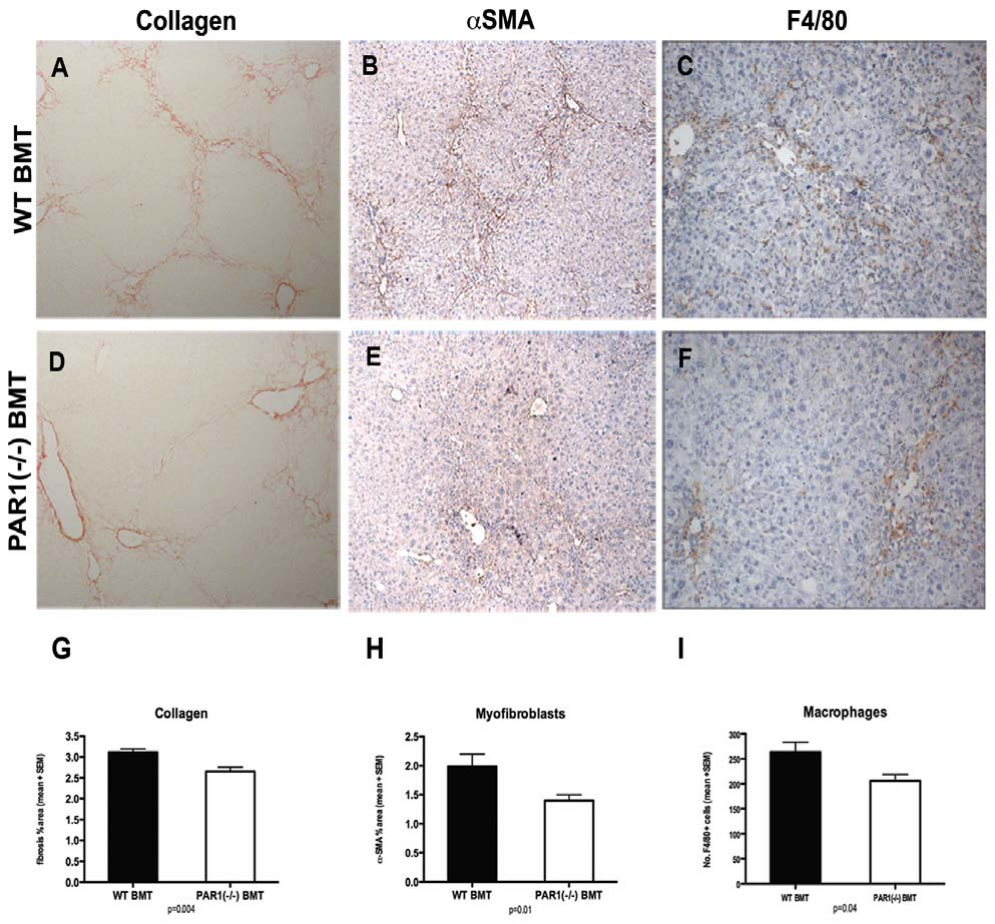
Absence of PAR1 signalling on BM-derived cells can confer protection against liver fibrosis. C57/BL6 WT mice transplanted with BM from PAR1 knockout donors (PAR1(−/−)BMT, D–F) acquired significantly less hepatic collagen (images A&D, sirius red, ×100; graph G), fewer activated myofibroblasts (images B&E, αSMA, brown, ×100; graph H) and fewer hepatic macrophages (images C&F, F4/80, brown, ×200; graph I) after CCl_4_ injury compared to control mice transplanted with WT BM from C57/BL6 donors (WTBMT, A–C). (graphs show mean + SEM, p values as shown.)

PAR1(−/−) BMT mice accumulated significantly less liver fibrosis than equivalent WT BMT controls (2.65+/−0.10% vs. 3.11+/−0.08%, collagen surface area, p = 0.004) after CCl_4_ injury, and had significantly lower density of activated myofibroblasts (1.40+/−0.10% vs. 1.99+/−0.21%, αSMA+ histological percentage surface area, p = 0.01, figure 5). Analysis of collagen at gene expression level revealed that PAR1(−/−) BMT mice had a trend to lower levels of COL1A1 mRNA compared to WT BMT controls though this difference did not reach statistical significance at this time-point of advanced fibrosis (p = 0.076) (figure 3). These data provide evidence that absence of PAR1 signalling on BM-derived cells can confer protection against CCl_4_-induced liver fibrosis, thus indicating that the BM can influence the liver's fibrotic response.

Analysis of macrophage recruitment showed that a considerable proportion of hepatic macrophages were BM-derived, with adjusted engraftment rates in WT mice of 60% (figure 4). Moreover, a significantly lower proportion of this population was of BM origin in PAR1(−/−) BMT mice (29.0+/−3.4% vs. 39.6+/−3.8%, unadjusted rates, p = 0.05), suggesting that PAR1 signalling may play a role in macrophage recruitment to the injured liver. A reduction in *total* hepatic macrophage number was also seen (206+/−13 vs. 264+/−23, p = 0.04, figure 5). Thus, PAR1 signalling on hepatic macrophages may influence hepatic macrophage recruitment during injury affecting total macrophage number.

### PAR1 activation induces macrophage migration but not proliferation

**Figure 6:**
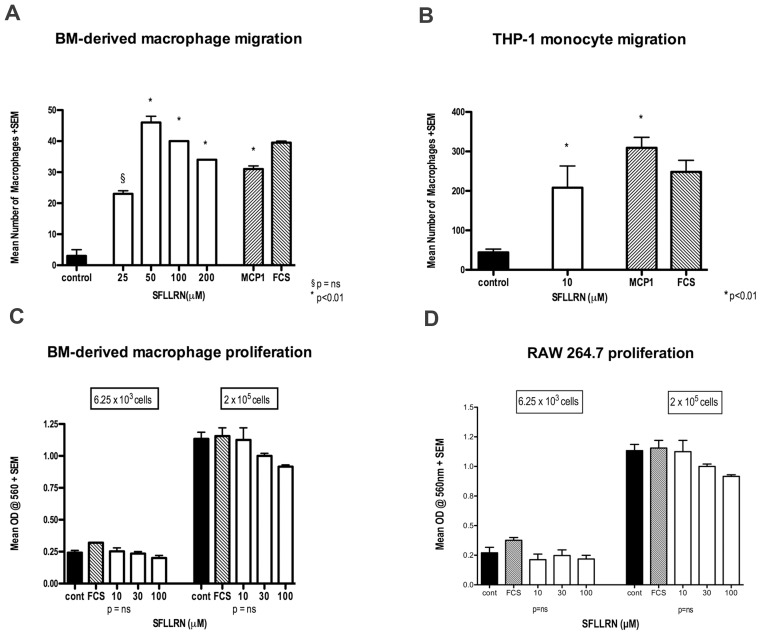
PAR1 activation induces monocyte/macrophage migration but not proliferation. (A–B) Migration assay of primary mouse BM-derived macrophages and a human monocyte cell line (THP-1) to SFLLRN demonstrating chemotaxis to the PAR1 agonist in both cell-types (MCP1 positive control). (C–D) SFLLRN has no pproliferative effect on primary mouse BM-derived macrophages (72 h) or a mouse macrophage cell line (RAW 264.7, 24 h) plated at the two different starting cell densities shown. (graphs show mean + SEM; p values as shown.)

The reduction of liver fibrosis in PAR1(−/−) mice was strongly associated with a reduction in hepatic macrophage numbers and found in conjunction with reduced macrophage recruitment to the liver from the BM. *In vitro* migration assays revealed that PAR1 activation *directly* induces macrophage/monocyte migration. Both primary BMDM's and the THP-1 human monocyte cell-line, demonstrated significant chemotaxis to the PAR1 ligand, SFLLRN (figure 6). In primary BMDM's the magnitude of the response was equivalent to that achieved with MCP1, a potent monocyte chemoattractant. In order to exclude the possibility that hepatic macrophage numbers were attenuated in PAR-1(−/−) BMT mice due to a direct effect of PAR1 stimulation on WT macrophage proliferation, primary BMDM's were incubated *in vitro* with SFLLRN. These studies revealed that PAR1 activation has no effect on macrophage proliferation (figure 6).

## Discussion

The importance of PAR1 signalling in liver fibrogenesis has been shown in murine models of cholestatic and parenchymal liver injury, and PAR1 expression in human liver disease has also been demonstrated [19], [22]–[24]. In this study using knockout mice, we confirm that PAR1 signalling plays an important role in CCl_4_-mediated liver fibrosis. We demonstrate that absence of PAR1 is associated with a 33% reduction in histological collagen deposition and a significant reduction in the activated αSMA-positive myofibroblast expansion. Our results are consistent with a previously published study where liver fibrosis reductions of 36% and 56% were reported in heterozygote and homozygote PAR1-deficient mice, respectively [24]. We furthermore go on to demonstrate that an important cell-type in PAR1 regulation of liver fibrosis is the hepatic macrophage. In our model of CCl_4_-induced liver fibrosis, we found that PAR1 expression in WT mice is highly up-regulated localising to both septal scars and the hepatic lobule itself. A large majority of the PAR1 expression seen within the injured liver occurred on hepatic macrophages. Scar-associated macrophages play an important role in releasing pro-inflammatory and pro-fibrogenic cytokines during liver injury [5]–[8]. Knockout of PAR1 in our study is associated with a decrease in macrophage number within the damaged liver. In other organ systems, loss of PAR1 signalling has been shown to be protective in a mouse model of crescentic glomerulonephritis in parallel with a decrease in renal macrophage number, and PAR-1 deficiency causes amelioration of bleomycin-induced lung injury in association with a reduction in macrophage recruitment to alveolar airspaces [13], [29].

We have gone on to demonstrate that BM transplantation alone can partially confer the phenotype of reduced hepatic fibrosis seen in PAR1(−/−) mice. In mice where only the BM was rendered PAR1-deficient (i.e. in WT mice transplanted with PAR1(−/−) BM), reductions in hepatic fibrosis, collagen gene expression and activated myofibroblast expansion were observed in conjunction with a diminution of the hepatic macrophage population. The impact on liver fibrosis seen here was of lower magnitude than that seen in wholly PAR(−/−) deficient, i.e. those not undergoing BM transplantation. This may reflect the fact that PAR1 signalling on cells endogeneous to the liver is also relevant. At the single time-point of advanced fibrosis studied in these experiments, only 30–45% of hepatic myofibroblasts were of BM origin, indicating chimerism in these cell populations in both PAR(−/−) and WT mice. Furthermore, up to 60% of the total hepatic macrophage population appeared BM-derived. Here, deficiency of PAR1 on BM cells led to a significant reduction in macrophage engraftment into the liver, suggesting a role for PAR1 signalling in macrophage recruitment. PAR1 signalling in other cells controlling inflammation and fibrosis, such as T-cell lymphocytes, may also play a role in liver fibrosis, but this has not been explored in these experiments [23], [24].

The importance of MCP1/CCL2 signalling to monocyte/macrophage chemotaxis is well described and recent studies have highlighted the importance of MCP1/CCL2 signalling via CCR2 receptors on BM-derived cells in the CCl_4_ mouse model of fibrotic liver injury [8], [30]. In these studies, mice deficient in CCR2 have reduced hepatic macrophage infiltration associated with reduced HSC activation and diminished liver fibrosis. It has previously been shown that PAR1 activation on rat HSCs induces MCP1/CCL2 synthesis *in vitro*, as well as stimulating HSC proliferation and collagen I expression [20], [22]. Likewise, PAR1 activation also stimulates MCP1/CCL2 production in both murine and human monocytes/macrophages [21], [31]. MCP1/CCL2-generated macrophage recruitment was shown to be dependent on PAR1 activation *in vivo* in a mouse heart to rat model of acute humoral rejection [32]. In the studies presented here, we have demonstrated that PAR1 activation can directly stimulate monocyte migration, independent of MCP-1 signalling, *in vitro*. This highlights a further pathway by which PAR1 signalling could recruit macrophages during liver injury to promote fibrosis. It is probable that several pathways exist by which PAR1 signalling can recruit hepatic macrophages to sites of injury. Conversely, we found that PAR1 activation did not promote macrophage expansion, suggesting that PAR1 signalling affects hepatic macrophage number predominantly via recruitment of these cell-types rather than their proliferation.

We sought to further test the hypothesis that the BM can play a significant role in liver fibrosis and have demonstrated this by selectively disrupting PAR1 signalling on BM-derived cells. We provide further evidence that BM-derived macrophages may play an important role in promoting liver fibrogenesis, and propose a role for PAR1-induced macrophage chemotaxis in this process.
